# Chemical Distributions of Different Sodium Hydroxide Molarities on Fly Ash/Dolomite-Based Geopolymer

**DOI:** 10.3390/ma15176163

**Published:** 2022-09-05

**Authors:** Wan Mastura Wan Ibrahim, Mohd Mustafa Al Bakri Abdullah, Romisuhani Ahmad, Andrei Victor Sandu, Petrica Vizureanu, Omrane Benjeddou, Afikah Rahim, Masdiyana Ibrahim, Ahmad Syauqi Sauffi

**Affiliations:** 1 Faculty of Mechanical Engineering Technology, Universiti Malaysia Perlis (UniMAP), Arau 02600, Perlis, Malaysia; 2 Center of Excellence Geopolymer & Green Technology (CeGeoGTech), Universiti Malaysia Perlis (UniMAP), Arau 01000, Perlis, Malaysia; 3 Faculty of Chemical Engineering Technology, Universiti Malaysia Perlis (UniMAP), Arau 01000, Perlis, Malaysia; 4 Faculty of Material Science and Engineering, Gheorghe Asachi Technical University of Iasi, 41 D. Mangeron St., 700050 Iasi, Romania; 5 National Institute for Research and Development in Environmental Protection INCDPM, Splaiul Independentei 294, 060031 Bucuresti, Romania; 6 Romanian Inventors Forum, St. P. Movila 3, 700089 Iasi, Romania; 7 Technical Sciences Academy of Romania, Dacia Blvd 26, 030167 Bucharest, Romania; 8 Department of Civil Engineering, College of Engineering, Prince Sattam Bin Abdulaziz University, Alkharj 16273, Saudi Arabia; 9 Department of Geotechnical and Transportation, Faculty of Engineering, Universiti Teknologi Malaysia, Johor 81310, Malaysia

**Keywords:** geopolymer, molarity, fly ash, dolomite, elemental distribution, compressive strength

## Abstract

Geopolymers are an inorganic material in an alkaline environment that is synthesized with alumina–silica gel. The structure of geopolymers consists of an inorganic chain of material and a covalent-bound molecular system. Currently, Ordinary Portland Cement (OPC) has caused carbon dioxide (CO_2_) emissions which causes greenhouse effects. This analysis investigates the impact on fly ash/dolomite-based-geopolymer with various molarities of sodium hydroxide solutions which are 6 M, 8 M, 10 M, 12 M and 14 M. The samples of fly ash/dolomite-based-geopolymer were prepared with the usage of solid to liquid of 2.0, by mass and alkaline activator ratio of 2.5, by mass. After that, the geopolymer was cast in 50 × 50 × 50 mm molds before testing after 7 days of curing. The samples were tested on compressive strength, density, water absorption, morphology, elemental distributions and phase analysis. From the results, the usage of 8 M of NaOH gave the optimum properties for the fly ash/dolomite-based geopolymer. The elemental distribution analysis exposes the Al, Si, Ca, Fe and Mg chemical distribution of the samples from the selected area. The distribution of the elements is related to the compressive strength and compared with the chemical composition of the fly ash and dolomite.

## 1. Introduction

Davidovits,1978 first coined the term geopolymer and patented it, and produced the geopolymerization reaction from fly ash [1]. The geopolymer does not form into calcium-silicate-hydrates (C–S–H) for matrix formation and strength but undergoes polycondensation process of silica and alumina to obtain structural capacity. Sodium hydroxide (NaOH) and sodium silicate (Na_2_ SiO_3_) are the two main ingredients in geopolymers. The main elements in the source materials are silicone (Si) and aluminum (Al) and should be thick and plentiful in aluminosilicate [2]. Geopolymers attract considerable attention due to their low costs, excellent mechanical and physical properties, low energy consumption and reduced greenhouse emissions.

Fly ash containing alumina-silicate (Al-Si) substance which has the ability to bind with calcium-hydroxide (CaOH_2_) in a cement matrix [3]. Fly ash is known as waste material from thermal power plants and its abundant availability creates an opportunity to research the uses of this material as a cement replacement. The main composition of fly ash contains silica (SiO_2_), aluminum oxide (Al_2_O_3_), iron oxide (Fe_2_O_3_) and calcium oxide (CaO) which are categorized as pozzolanic materials. Because of the pozzolanic reaction and its microfiller feature, fly ash could enhance the properties of cement and concrete [4]. Fly ash also can enhance the workability and mechanical properties in terms of compressive strength, flexural strength, as well as the toughness of cement. Singh N. [5] reported that the production of geopolymer cement and concrete using fly ash as alternatives to the OPC improves the durability in acidic environments and gave enhanced densification when the fly ash geopolymer is cured in a microwave. Sitarz et al. [6] studied how the addition of Ground granulated blast furnace slag (GGBFS) to fly ash (FA)-based geopolymers affects the evolution of the strength of the resultant geopolymer mortars. Results found that blending of GGBS and FA improved the mechanical properties of compressive and flexural tensile strength with the highest strength achieved being fc = 63 MPa and ft = 6.8 MPa at 28 days of curing.

Dolomite is a type of volcanic rock that is particularly resistant to the weight of objects and is also dispersible. Because of its increased surface toughness and thickness, dolomite is suitable for use in the building industry [7]. In the geopolymer field, the use of dolomite as a precursor remains recent and studies are still ongoing. Dolomite often has a variety of natural minerals, which are mostly inexpensive and are chosen because of their strong magnesium oxide content. Dolomite is a primary sediment mineral and is widely distributed geologically. Calcium carbonate minerals, such as dolomite are also usually applied to geopolymers to improve the mechanical properties of geopolymers [8]. Coppola et al., [9] investigated the role of composition and processing of carbonate-containing alkali activated materials, such as granite, albite, diatomite combines with metakaolin and fly ash. The review observed the development of alkali-activated materials using natural stone waste and minerals increases the workability of the fresh mixtures and the mechanical and durability properties of the hardened materials.

As a geopolymer binder needs to dissolve both silica (Si) and alumina (Al) oxides in an alkaline medium, alkali activator solutions perform an important role to produce geopolymer materials. The composition of the activator has a significant effect on the compressive strength of the geopolymer materials [10]. Sodium hydroxide (NaOH) and sodium silicate (Na_2_ SiO_3_) are the most effective alkaline solutions due to the chemical characteristics of the compound. Kumar et al. [11] demonstrated that the use of Na_2_SiO_3_ solution with NaOH solution increases the rate of the geopolymerization reaction compared to the use of NaOH alone. Besides, the NaOH solution also provides a wider range of dissolution than the potassium hydroxide (KOH) solution. In this analysis, the NaOH molarity is examined to determine its impact on the properties of the fly ash/dolomite-based geopolymers and the elemental distribution in geopolymer materials.

## 2. Materials

### 2.1. Solid Precursor

Fly ash class C is used as a raw material for this analysis. The raw material was gathered in Manjung Power Station, Lumut, Perak, Malaysia. Fly ash is a thinly isolated mineral material from coal-fired power plants. Fly ash consists of an inorganic, non-combustible substance, formed in a glassy amorphous state after combustion. The form and size of fly ash particles are typically 2 μm to 10 μm. The major composition of fly ash class C, which is described in Table 1, is SiO_2_, which is about 30.7% of the total composition, and the smallest is TiO_2_, which is around 0.94% of the total composition. 

Dolomite is a natural resource consisting of calcium and magnesium carbonate and containing iron. Compared to fly ash, dolomite has low Al + Si such that dolomite cannot stand alone. Table 1 includes CaO, which accounts for approximately 74.26% and MgO for approximately 21.42% of the overall composition. Dolomite was shipped to Universiti Malaysia Perlis from Quarry Perlis. Before the chemical composition examination, the dolomite must be browned and tamed on a scale from 75 μm to 63 μm. Table 1 shows the chemical compositions of Fly ash C and Dolomite used in this study.

### 2.2. Alkaline Activator

Sodium hydroxide (NaOH) and sodium silicate (Na_2_SiO_3_) were used as the alkaline activator solution. NaOH is typically in pallet form and dissolved with distilled water to produce NaOH solution with certain molarities. Na_2_SiO_3_ solution is a glassy substance that is soluble in water and has a very valuable quality.

## 3. Methodology

### 3.1. Preparation of Sodium Hydroxide (NaOH) Solution

The NaOH solution was prepared by dissolving the NaOH pallets with distilled water until achieved homogeneity. The liquids were stirred and cooled at room temperature until the solutions become clear. The NaOH solution was prepared at 6 M, 8 M, 10 M, 12 M and 14 M concentrations according to Equation (1) below:

(1) $$\begin{array}{cc} & \mathrm{Mass}\;\mathrm{of}\;\mathrm{NaOH}\;\mathrm{used}\;=m=\frac{\mathrm{mv}}{1000}\times \left(\mathrm{molecular}\;\mathrm{weight}\;\mathrm{of}\;\mathrm{NaOH}\right)\\ = & \frac{\left(12\times 1000\right)}{1000}\times \left(23+16+1\right)\;g/\mathrm{mol}\;=\left(12\;\mathrm{mol}\right)\left(40\;g/\mathrm{mol}\right)=480\;g \end{array}$$

### 3.2. Sieving Process

The sieving process is a system for deciding the particle size distribution of the sample. This study includes the sieve method for dolomite content with a scale of approximately 150 μm to 63 μm. The sieving time is measured at about 60 min. The procedure is replicated until the appropriate dolomite particle size exceeds.

### 3.3. Mixing Process

The fly ash and dolomite were mixed with 80% of fly ash and 20% of dolomite. After, the alkaline activator solutions were added to the fly ash/dolomite mixture with a ratio of 4.0 by mass. The alkaline activator solutions of combinations of NaOH and Na_2_ SiO_3_ solution were mixed first with a constant ratio of 2.5, by mass before combining with the fly ash/dolomite mixture. Using a mechanical mixer, the mixture of fly ash/dolomite and alkaline activator solutions was mixed for 5 min and then placed into a 50 × 50 × 50 mm mold.

### 3.4. Curing Process

For this study, the fly ash-based geopolymer was cured at room temperature only because class C fly ash has a characteristic of self-hardening and benefits from being hardened without using a high temperature. The fly ash/dolomite-based geopolymer was cured for 7 days prior to testing. After 7 days of curing, the samples were removed and tested for compressive strength, water absorption, density and microstructure analysis.

### 3.5. Compressive Strength Test

The most significant property of cement and concrete is the compressive strength test. The strength of fly ash/dolomite-based geopolymer was tested at 7 days of curing by using a compression test machine Instron machine series 5569 Mechanical Tester. The results were recorded in N/mm2. The standard used for this testing was ASTM C109.

### 3.6. Water Absorption Analysis

The fly ash/dolomite-based geopolymer were tested for water absorption in a cube form of 50 × 50 × 50 mm at 7 days of aging. A water absorption test was performed according to ASTM C 140. Equation (2) below specifies the calculation for water absorption analysis:

(2) $$\mathrm{Water}\;\mathrm{absorption}\;=\left[\frac{\left(\mathrm{Wet}\;\mathrm{weight}-\mathrm{Dry}\;\mathrm{weight}\right)}{\mathrm{Dry}\;\mathrm{weight}}\times 100\right]$$

### 3.7. Density Analysis

The fly ash/dolomite-based geopolymer density was measured at room temperature after 7 days of aging. The density of geopolymer samples was recorded based on Equation (3) below:

(3) $$\mathrm{Density}\;=\frac{\mathrm{mass}\;\mathrm{of}\;\mathrm{the}\;\mathrm{sample}\;\left(\mathrm{kg}\right)}{\mathrm{volume}\;\mathrm{of}\;\mathrm{the}\;\mathrm{sample}\;\left(m^{3}\right)}$$

### 3.8. Porosity Analysis

The porosity of fly ash/dolomite-based geopolymer was determined in accordance with ASTM C642 [12] and calculated using the following formula, Equation (4).

(4) $$\mathrm{Porosity}\;\left(\%\right)=\frac{\mathrm{Wet}\;\mathrm{weight}-\mathrm{Dry}\;\mathrm{weight}}{\mathrm{Wet}\;\mathrm{weight}-\mathrm{Suspended}\;\mathrm{weight}}\times 100$$

## 4. Results and Discussion

### 4.1. Compressive Strength Analysis

**Figure 1:**
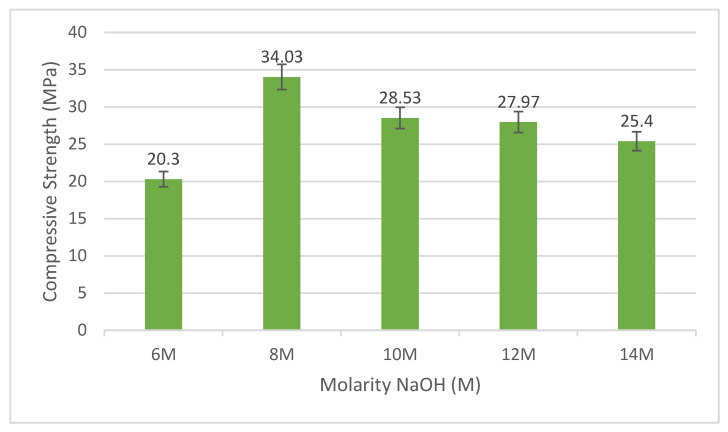
Fly ash/dolomite geopolymer compressive strength.

Figure 1 indicates the compressive strength of the various NaOH molarity of fly ash/dolomite-based geopolymer. The strength of fly ash/dolomite-based geopolymer increases from 20.3 MPa to 34.03 MPa when the molarity increases from 6 M to 8 M. However, the strength decreases from 34.03 MPa to 25.4 MPa when the molarity of geopolymer was increased up to 14 M. The increment of the strength from 6 M to 8 M was due to the occurrence of Na^+^ ions and enough OH^−^ ions supplied by the source materials to be bonded with the alkaline solutions. This was due to the role of both Na^+^ ions and OH^−^ ions in the geopolymer systems to dissolve the bond of the source materials (Si and Al bond) to produce Si–O–Al bonds which are known as geopolymer precursors. The Na^+^ ions were responsible for balancing the negative charges produced from the formation of Si–O–Al bonds while the OH^−^ ions played a vital role in the hydrolysis process of the geopolymer, producing a good reaction and bonding which led to better compressive strength [13].

**Figure 2:**
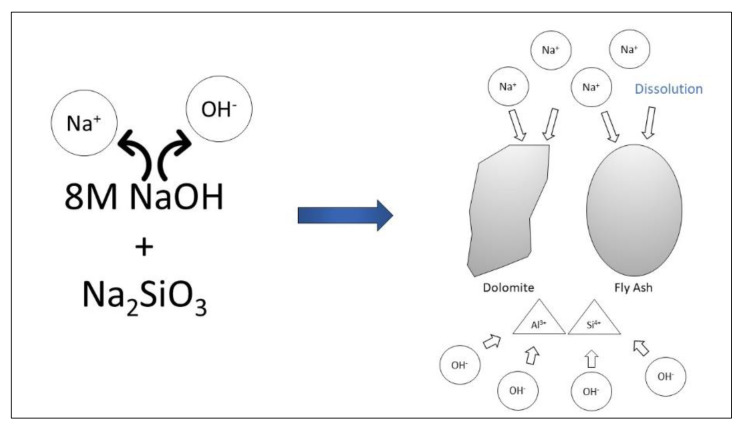
Ion balance and dissolution process in the dolomite/fly ash based geopolymer.

The 8 M of NaOH centered on fly ash/dolomite-based geopolymer demonstrates optimum intensity, due to the improved shape, reaction and efficient geopolymer formulation between the dolomite and alkaline activator. This is because 8 M NaOH molarity could provide enough ions of Na and OH to react with the fly ash and dolomite to release Al^3+^ and Si^4+^ ions. The optimum NaOH concentration contains adequate Na^+^ ions content which makes the fly ash and dolomite composition uniform, thus improving the geopolymerization rate by balancing charges as well as forming an aluminosilicate network. This is proved by Khale and Chaudhary [14] that found the accurate rate of NaOH concentration will form great dissolution of silica and alumina from the raw materials thus directing to improved strength. Figure 2 shows the illustration of the ion balance and dissolution process in the dolomite/fly ash based geopolymer. The Na^+^ ions were needed for the dissolution of the geopolymer material to release Al^3+^ and Si^4+^ ions, meanwhile the OH^−^ ions will react with the released ion during the geopolymerization process. Kiatsuda Somna et al. studied the stand-alone fly ash geopolymer with 4.5–16.5 M NaOH used. With focus on strength, the compressive strengths at 28 days of 20.0–23.0 MPa were obtained with NaOH concentrations of 9.5–14.0 M. Increasing the NaOH concentration beyond this point resulted in a decrease in the strength of the paste due to the early precipitation of aluminosilicate products [15]. This shows the same pattern as the finding in this experiment.

However, with NaOH concentrations of 10 M to 14 M, the fly ash/dolomite compressor capacity becomes slow. This was due to the excess of Na^+^ and OH^−^ ions from the high molarity of NaOH used. The unnecessary OH^−^ ions in the geopolymer cause early precipitation of aluminosilicate gel. These will disturb the process of releasing the Al^3+^ and Si^4+^ ions in the fly ash and dolomite and cause the geopolymerization rate to be lowered as well, producing a low compressive strength of geopolymer [16]. This was also due to the high Ca content in dolomite which affects the gelation of silica (Si) during geopolymerization process and distracts the compressive strength output [17].

### 4.2. Density Analysis

**Figure 3:**
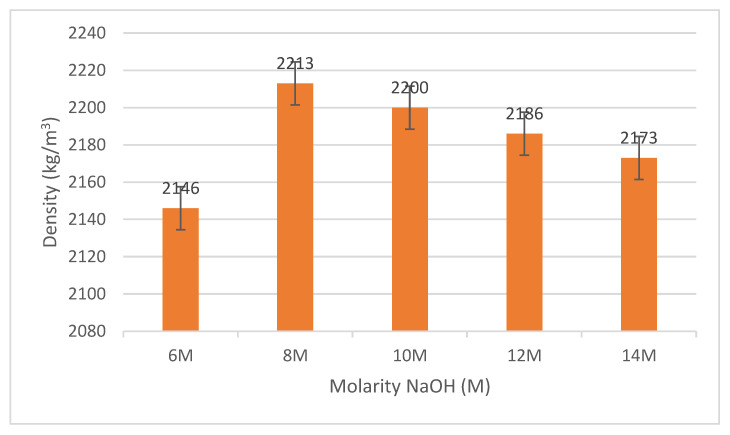
Density of fly ash/dolomite geopolymer.

The fly ash/dolomite-based geopolymer densities of different NaOH solution molarities are shown in Figure 3. Densities ranged from 6 M to 14 M between 2140 kg/m^3^ and 2220 kg/m^3^, respectively. After 7 days, the maximum density value was up to 2213 kg/m^3^. The concentration of NaOH influences the fly ash/dolomite geopolymer properties. Results showed that increased NaOH concentration typically increases the geopolymer density. The increase in NaOH molarity from 6 M to 8 M reveals a decrease in porosity. Fly ash/dolomite geopolymer with the least molarity is more permeable, so it has a lower density than fly ash/dolomite with higher molarity [18]. As the concentration of NaOH increases, workability, water absorption and the density of geopolymer would be decreased [19].

It may also be triggered by a curing phase at room temperature which allowed the atmosphere to fluctuate the samples. The increase in density was also attributed to the improvement in the sample viscosity in the solution with a high NaOH concentration. The NaOH mixtures on 8 M can be seen to be more coherent. The denser the sample, the stronger the compressive strength; an adequate alkaline activator is essential [20]. Normally the density of the geopolymer is parallel to the strength of the geopolymer. This was due to the 8 M NaOH solution producing a greater dissolution process of Si and Al during the geopolymerization process [21].

### 4.3. Water Absorption Analysis

**Figure 4:**
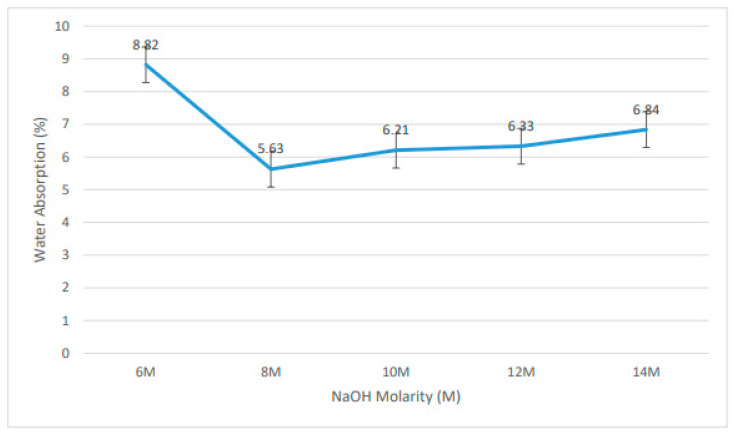
Rate of water absorption for fly ash/dolomite geopolymer.

Figure 4 shows an analysis of geopolymer water absorption dependent on fly ash/dolomite-based geopolymer. The 8 M NaOH usage produced the lowest water absorption rate at 5.63%, and the highest water absorption was at 6 M NaOH with 8.82%. This result shows the relationship between the NaOH concentration and the porosity of the geopolymer samples. Pores are used to measure the penetration of water in the geopolymer samples. Pores within geopolymer samples are formed based on several conditions and parameters, such as the consistency of water, binding strength, and treatment techniques [22].

As the compressive strength of the geopolymer decreases, the water density increases. A high degree of water retention instigated the lowest compressive strength and porosity [23]. This is because the influence of the microstructure of the fly ash/dolomite geopolymer on the C–S–H (Ca–Si–H) system is much coarser and permeable, which clarifies the reason for increased water assimilation and decreased characteristics of the geopolymer samples [24]. The low water absorption is a strong indication that open porosity decreases and impedes the heavy flow of water into the geopolymer portion. The lower water absorption by the geopolymer samples means that the samples are durable [25].

### 4.4. Porosity Analysis

**Figure 5:**
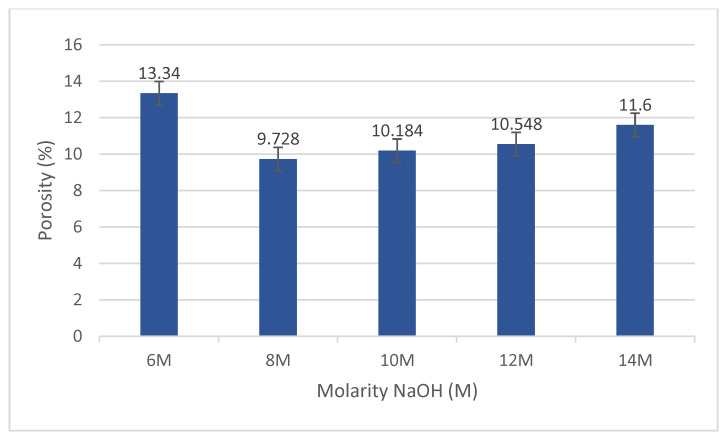
Porosity of fly ash/dolomite geopolymer.

Figure 5 depicts the apparent porosity of the fly ash/dolomite geopolymer with varying NaOH molarities. Overall, the porosity results follow the pattern of the water absorption results, as samples with higher water absorption tend to have greater porosity content.

The fly ash/dolomite geopolymer porosity is made up of both entrained air voids and internal paste voids. According to Figure 5, increasing the sodium hydroxide concentration from 10 M to 14 M caused the fly ash/dolomite geopolymer microstructure to become significantly coarser and porous, which accounts for the increased porosity and decreased strengths at higher NaOH concentrations. As claimed by Gorhan and Kurklu, [26] increasing the sodium hydroxide concentration increased apparent porosity due to an increase in viscosity and water evaporation during the polymerization process, which resulted in linked pores, a high tendency for permeability and increase porosity. The ideal NaOH concentration in this investigation for making fly ash/dolomite geopolymers was 8 M with 9.72% apparent porosity. Increased density at 8 M NaOH concentration means less porosity and water absorption, which contributes to higher compressive strength. In contrast to Patankar et al. [27], determined 12 M to be the optimal concentration for the NaOH solution; the concentration of NaOH solution determined by this study was lower. It might be owing to the varied formulation of geopolymer mixtures.

### 4.5. Morphology Analysis

**Figure 6:**
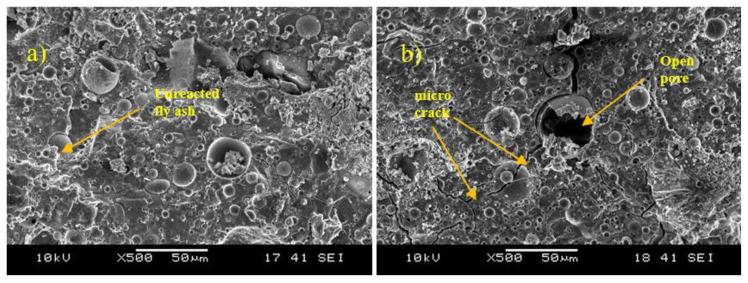
Fly ash/dolomite morphology for (**a**) 8 M and (**b**) 14 M NaOH Molarity.

The morphology of the fly ash/dolomite-based geopolymers focuses on the 8 M and 14 M NaOH concentrations. This is because the optimum molarity used for this study is 8 M and the lowest performance appeared at 14 M. The morphology structure was performed using the Scanning Electron Microscope (JEOL) platform JSM-6460 LA Japan. Figure 6 displays the comparison morphology structure between 8 M and 14 M concentrations of NaOH.

From the morphology analysis, it can be observed that the 8 M of NaOH produces a better surface compared to the 14 M NaOH concentration. The 14 M NaOH surface tends to create cracks along the samples. These cracks caused the flow of water and retarded the formation of a bond between the raw materials and the alkaline activator solutions. Besides that, there is also some unreacted fly ash on both samples. This was due to the curing conditions of the samples which are not enough to produce the geopolymer gel [28]. Longer curing days will affect the geopolymerization rate and reduce the unreacted fly ash hence improving the strength of the geopolymer samples.

This morphology analysis strengthens the findings on compressive strength which indicates that an 8 M NaOH concentration provides a better interaction between the fly ash and dolomite which leads to a good polymerization reaction and better strength. The cracks and pores observed for the 14 M NaOH concentration cause disturbances in the geopolymer, and in turn, a less dense geopolymer product that can withstand less strength. Besides that, 14 M NaOH concentration led to excessive Na^+^ ions in the geopolymer system causing the geopolymerization to be incomplete, thereby creating the pores and cracks that can be observed in the morphology [15].

**Figure 7:**
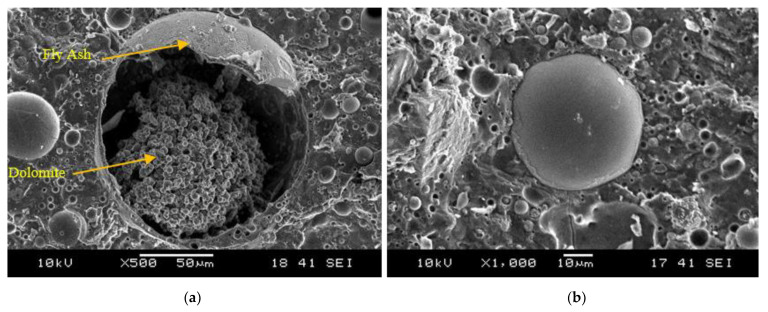
The morphology of (**a**) reaction between fly ash and dolomite and (**b**) gelation process of the geopolymer.

Figure 6 shows the close-up microstructure of the samples for fly ash/dolomite geopolymer. It clearly illustrates the reaction between fly ash and dolomite. Figure 7a shows the reaction that occurred between fly ash and dolomite within the fly ash particles. The fly ash particles are in a sphere shape meanwhile the dolomite has an irregular cloud shape [29]. Figure 7b elucidates the gelation process of the geopolymer which is the Ca–S–H gel that is formed during the geopolymerization process.

**Figure 8:**
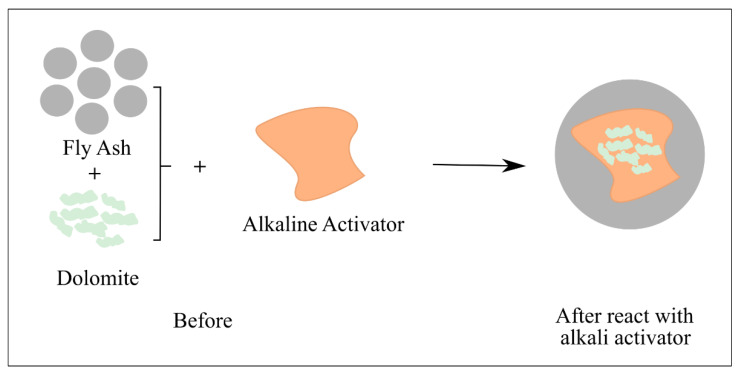
Schematic diagram of the reaction between fly ash and dolomite geopolymer.

As demonstrated in Figure 8, a gel network underwent the geopolymerization rearrangement process after the alkali activator reacted with fly ash and dolomite. This stimulated the formation of the geopolymer matrix, while the dolomite filled the space within the geopolymer structure.

### 4.6. Elemental Distribution Analysis

**Figure 9:**
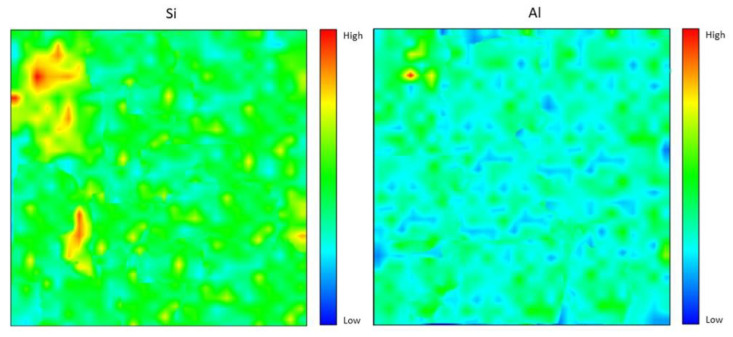
Si and Al elemental distribution for 8 M of NaOH fly ash/dolomite geopolymer.

The element observed for the geopolymer samples were Silica (Si), Alumina (Al), Calcium (Ca), Magnesium (Mg) and Iron (Fe). The elements were chosen based on the major components in fly ash and dolomite. The indicator was identified in the distribution analysis results to determine the availability of the element in the geopolymer samples. The indicator shows red for high intensity, green for medium intensity, and blue for low intensity. Figure 9 demonstrates the Si and Al elemental distribution for the fly ash/dolomite geopolymer with an 8 M NaOH concentration used. The Si element is well distributed all over the geopolymer samples. There were a few spots that show a high peak of Si element detected in the result. As for the Al element, it was also distributed well all over the geopolymer samples. However, the intensity of the Al element was slightly lower compared to the Si element. The color of the intensity for the Si element is greener compared to the Al element, which is a slightly blue color. This result is reflected in the chemical composition results from Table 1. Based on the results, the composition of Si is higher compared to the composition of Al which mainly comes from fly ash [30].

**Figure 10:**
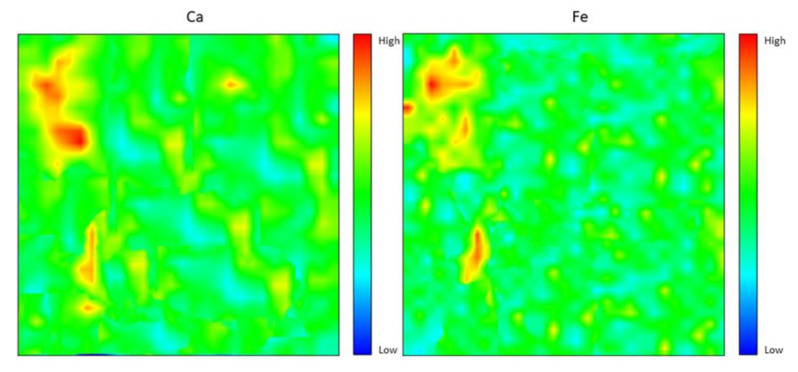
Ca and Fe elemental distribution for 8 M of NaOH fly ash/dolomite geopolymer.

Figure 10 shows the elemental distribution for the Ca element and Fe element. Both distributions were scattered appropriately. There were slight differences between the Ca and Fe elemental distribution, which was that Ca has a larger distribution with green intensity compared to Fe. Besides that, there were some places that Ca reached high intensity with red color at a few spots, which is almost the same spot as the Fe element. Other than that, based on the chemical composition analysis, Ca has the highest composition, which comes from fly ash and dolomite. The Fe content was only contributed to by fly ash, thus resulting in less elemental distribution. It can be observed that the Ca distribution is higher compared to the Si and Al distribution; [31] concluded that high calcium fly ash (which contains high Ca content) has the potential to be used as the source material for geopolymer synthesis with self-hardening characteristics which support the production of high strength geopolymer [32].

**Figure 11:**
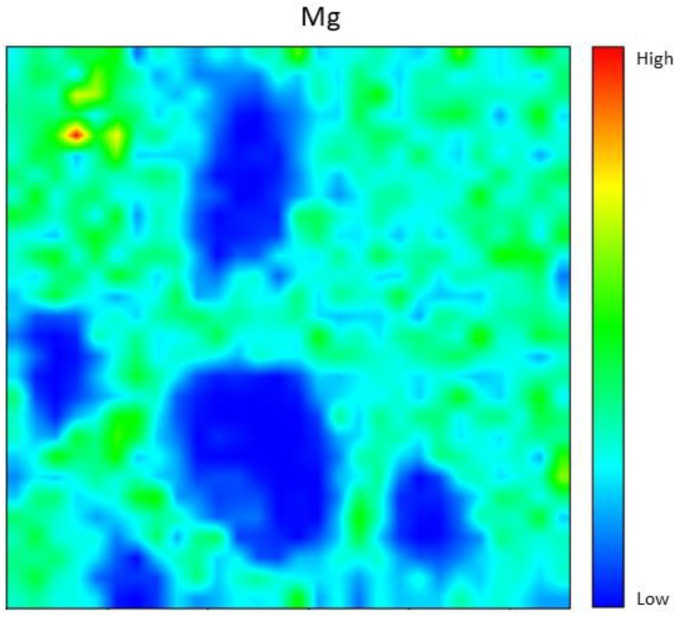
Mg elemental distribution for 8 M of NaOH fly ash/dolomite geopolymer.

Figure 11 shows the elemental distribution of Mg and the results obtained show that Mg was a bit different compared to other elements. As seen in the figure, few spots appeared which indicated the low intensity of the element. This happened because the Mg content only came from 20% of dolomite samples. The Mg was too little to react with other elements and only react with a few elements producing tiny spots of elemental distribution. However, the Mg element could still contribute to the strength of the geopolymer with moderate composition [33].

Generally, elemental distribution results show a good spread of elements all over the geopolymer samples and produced high strength geopolymer samples with 8 M concentrations. The geopolymer’s main structure consists of Si–O–Al, which clearly shows the importance of Si and Al elements in producing good strength development [32,34,35,36]. Well-distributed elements in the geopolymer composite samples indicated the production of a homogenous geopolymer composite. A homogenous mixture of geopolymer composites enhances the strength of the material. The combination of Ca, Si, and Al maps led to the formation of a calcium aluminate hydrate (C-A-S-H) phase which leads to the development of strength with self-hardening characteristics at room temperature [37].

### 4.7. Phase Analysis

**Figure 12:**
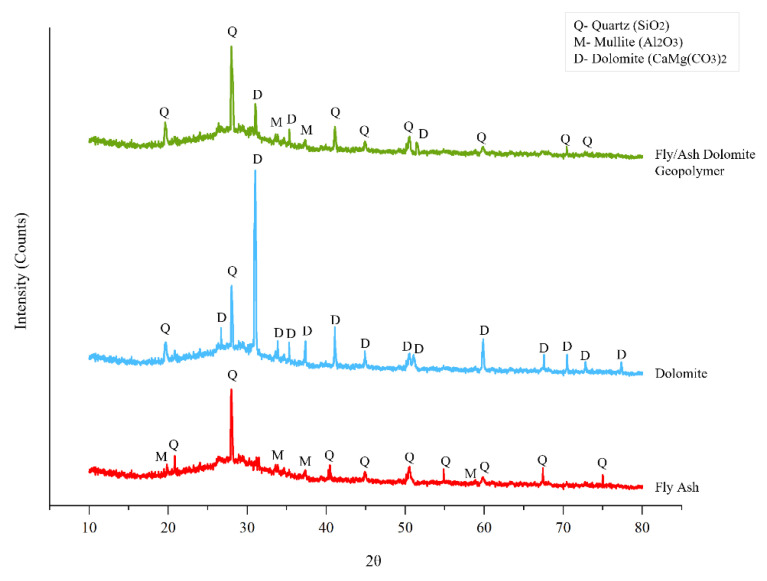
XRD patterns of fly ash, dolomite and fly ash/dolomite geopolymer.

Figure 12 shows the comparison of phase analysis composition of fly ash, dolomite and fly ash/dolomite geopolymer with 8 M NaOH concentrations that were evaluated through X-ray powder diffraction analysis (XRD). The phase that can be observed were mainly quartz (SiO_2_), mullite (3 Al_2_ O_3_(2 SiO_2_)) and dolomite (CaMg(CO_3_)_2_).

The highest peak observed on fly ash was quartz meanwhile on dolomite it was the dolomite phase. The combination both of materials that produce a geopolymer after being activated by alkaline solutions at 8 M NaOH concentration created a high phase of quartz and dolomite. There is a huge “amorphous hump” that rises between 20° to 40° degrees 2θ which is illustrative of a geopolymeric reaction.

From the XRD patterns of fly ash, the peaks indicate quartz (SiO_2_) (ICDD: 00-0461-045) as a major crystalline peak at 2θ values of 27.993°. Less intense quartz was also observed at 2θ values of 20.836°, 40.479°, 44.484°, 50.535° and 67.42°. The peak of mullite (3 Al_2_ O_3_(2 SiO_2_)) (ICDD 00-015-0776) was obtained at 19.835°, 33.85°, 35.296° and 58.877°. According to the findings of Thamer Alomayri [38], amorphous aluminosilicates alone in fly ash are reactive in the geopolymer formation process. Aside from that, the crystalline phases with primary peaks of the dolomite raw sample were indexed with the dolomite (CaMg(CO_3_)_2_) (ICDD 00-036-0426) reference pattern at 2θ values of 31.002°, 28.022°, 33.85°, 35.296°, 37.365°, 41.080°, 50.513° and 59.879°.

Geopolymerization reaction requires Si and Al which are mainly detected in this phase analysis. Therefore, the quartz phase exhibited as a major mineral shows a significant peak for fly ash/dolomite geopolymer at 2θ values of 27.993°, 19.612°, 41.105° and 50.446° which shows the availability of Si in the geopolymer system that mainly came from the fly ash. Meanwhile, the mullite phase as minor minerals was also observed at peak 2θ values of 33.583° and 37.365°. In line with Saliha Alehyen et al., the mullite phase or alumina silicate shows the availability of amorphous silica and alumina in the fly ash/dolomite geopolymer [39]. The dolomite phase was also detected in the fly ash/dolomite geopolymer. However, the phase was not as high as the quartz and mullite phases. This was due to the usage of dolomite in the geopolymer was minor (filler) compared to the fly ash.

## 5. Conclusions

Based on the analysis performed, the sodium hydroxide (NaOH) molarity plays an important role in the fly ash/dolomite-based geopolymers properties in terms of compressive strength, water absorption, density, and the elemental distribution of geopolymer samples. The fly ash/dolomite-based geopolymer contributed to the optimum NaOH concentration at 8 M based on the performance obtained from this research. The microstructure of the geopolymer samples provides an appearance of homogeneity and includes fewer unreacted fly ash and dolomite microspheres. The elemental distribution analysis was performed at optimum concentration and proved when 8 M of NaOH was used, the distribution of Si, Al, Ca, Mg and Fe elements are clearly formed and well distributed in the samples. This contributed to the development of strength without using a high temperature and was produced with a simple method at room temperature. The cure time often plays a key role in the compressive intensity of the geopolymer samples, and thus 24 h of cure time at room temperature gives an optimum time for the good performance of fly ash/dolomite geopolymer samples. Further studies need to be explored to find the benefits of minerals in dolomite and to produce them for wide applications.

## Figures and Tables

**Table 1:** The composition of fly ash class C and Dolomite.

Compound	Mass of Fly Ash Class C (wt.%)	Mass of Dolomite (wt.%)
SiO_2_	30.70	2.19
Al_2_O_3_	13.30	1.09
MgO	3.60	21.42
CaO	22.40	74.26
Fe_2_ O_3_	23.92	0.22
TiO_2_	0.94	0.06
SO_3_	2.28	0.85
Na_2_O	-	1.26

## Data Availability

Not applicable.
